# Supplementary material for: Splice-Junction-Based Mapping of Alternative Isoforms in the Human Proteome
Source: Cell Rep. Author manuscript; Available in PMC 2020 Jan 15. (PMC6961840; doi:10.1016/j.celrep.2019.11.026)
Supplement: 3 [file NIHMS1546469-supplement-3.zip › DF2/PXD009737/TestisGluC-101-Q8NCE2-LVPAGAPTQAAWLAALSDRE.pdf]

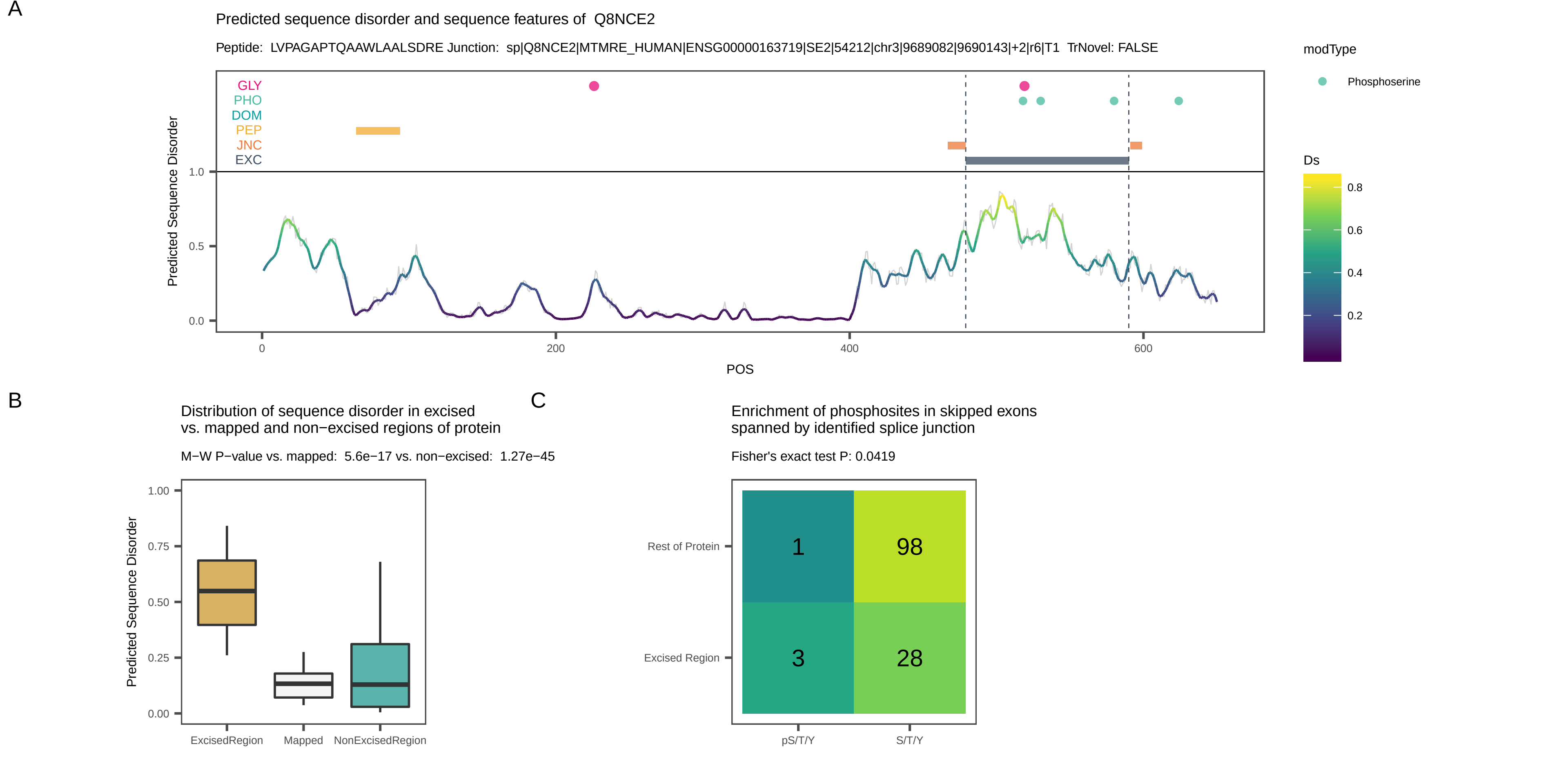
A
Predicted sequence disorder and sequence features of Q8NCE2
Peptide: LVPAGAPTQAAWLAALSDRE Junction: sp|Q8NCE2|MTMRE_HUMAN|ENSG00000163719|SE2|54212|chr3|9689082|9690143|+2|r6|T1 TrNovel: FALSE
modType
Phosphoserine
GLY
PHO
DOM
PEP
JNC
EXC
Predicted Sequence Disorder
1.0
0.5
0.0
0
200
400
600
POS
Ds
0.8
0.6
0.4
0.2
B
Distribution of sequence disorder in excised vs. mapped and non−excised regions of protein
M−W P−value vs. mapped: 5.6e−17 vs. non−excised: 1.27e−45
Predicted Sequence Disorder
1.00
0.75
0.50
0.25
0.00
ExcisedRegion
Mapped
NonExcisedRegion
C
Enrichment of phosphosites in skipped exons spanned by identified splice junction
Fisher's exact test P: 0.0419
Rest of Protein
Excised Region
1
98
3
28
pS/T/Y
S/T/Y
